# Standardized image quality for ^68^Ga-DOTA-TATE PET/CT

**DOI:** 10.1186/s13550-020-0601-y

**Published:** 2020-03-23

**Authors:** Christina P. W. Cox, Marcel Segbers, Laura H. Graven, Tessa Brabander, Daniëlle M. E. van Assema

**Affiliations:** Department of Radiology & Nuclear Medicine, Erasmus Medical Center, Postbus 2040, 3000 CA Rotterdam, The Netherlands

**Keywords:** Image quality, Neuroendocrine tumor, PET/CT, ^68^Ga-DOTA-TATE, Dose regimen, Optimizing, Body mass

## Abstract

**Background:**

Positron emission tomography (PET) imaging with ^68^Gallium labeled somatostatin analogues (^68^Ga-DOTA-SSA) plays a key role in neuroendocrine tumor management. The impact of patient size on PET image quality is not well known for PET imaging with ^68^Ga-DOTA-SSA. The aim of this study is to propose a dose regimen based on patient size that optimizes image quality and yields sufficient image quality for diagnosis.

**Methods:**

Twenty-one patients (12 males, 9 females) were prospectively included for ^68^Gallium-DOTA-Tyr3-Octreotate (^68^Ga-DOTA-TATE) PET/CT, which was acquired in whole body list mode using 6 min per bed position (mbp). The list-mode events were randomly sampled to obtain 1 to 6 mbp PET reconstructions. For semi-quantitative assessment of image quality, the signal-to-noise ratio (SNR) was measured in the liver. The SNR normalized (SNRnorm) for administered activity and mbp was correlated with body mass, length, body mass index, body mass/length, and lean body mass. Three experienced nuclear medicine physicians visually graded image quality using a 4-point scale, and categorically scored the number of somatostatin-receptor positive lesions for each reconstruction. To investigate the impact of image quality on lesion quantification, the mean, maximum, and peak standardized uptake values (SUVs) of one abdominal lesion were measured in the 1 to 6 mbp PET reconstructions.

**Results:**

Of all patient-dependent parameters, body mass showed the strongest correlation (*R*^2^ = 0.6) with SNRnorm. Lesion detectability analysis showed no significant difference for 3-5 mbp compared with the complete 6 mbp PET reconstruction. The SUV measurements showed no significant (*p* > 0.05) differences across the reconstructions. Visual assessment revealed that an SNR of 6.2 results in PET scans with moderate to good image quality. A non-linear expression was derived to calculate the required (dose × acquisition time) product (DTP) for the chosen SNR level of 6.2 that would yield a more constant image quality.

**Conclusion:**

Body mass can be used to predict ^68^Ga-DOTA-TATE PET image quality. The proposed non-linear dose regimen based on body mass standardizes the image quality while maintaining sufficient image quality for diagnosis.

## Background

Neuroendocrine tumors (NETs) are a heterogeneous group of tumors, originating from neuroendocrine cells, and are mostly located in the small intestine, pancreas, and lungs. The majority of these tumors have an overexpression of somatostatin receptors (SSTR) on their cell membrane. Radiolabeled somatostatin analogues (SSA) with high affinity for SSTR subtype 2 enable in vivo imaging of NETs as well as peptide receptor radionuclide therapy (PRRT) [1, 2]. For years, planar imaging and single photon emission computed tomography (SPECT) with ^111^In-pentetreotide (^111^In-DTPA^0^-octreotide (OctreoScan, Mallinckrodt St Louis, USA)) was the most important nuclear medicine imaging technique for diagnosis and treatment management of NETs [3, 4]. However, in recent years, positron emission tomography/computer tomography (PET/CT) imaging with Gallium-68 labeled SSA has become widely available.

The Erasmus Medical Center uses ^68^Gallium-DOTA-Tyr3-Octreotate (^68^Ga-DOTA-TATE), which predominantly binds to SSTR subtype 2 [4, 5]. Its theranostic twin ^177^Lutetium-DOTA-tyr3-Octreotate (^177^Lu-DOTA-TATE) is a well-established treatment for NETs [6, 7]. Other commonly used tracers are ^68^Gallium-DOTA-D-Phe1-Tyr3-Octreotide (^68^Ga-DOTA-TOC) [8], which also binds to SSTR subtype 5 and ^68^Gallium-DOTA-1-Nal3-Octreotide (^68^Ga-DOTA-NOC) [9], which binds to SSTR subtypes 2, 3, and 5.

PET imaging provides a higher spatial resolution and sensitivity compared with SPECT imaging, resulting in better image quality with shorter acquisition times [4, 5, 8, 10]. PET image quality depends, among others, on the quality of the PET camera, the tracer distribution, the amount of administered activity, and the acquisition time used for scanning [11, 12]. Image quality is also influenced by patient size, due to a variable amount of attenuation within patients with different habitus [11, 12]. The administered activity should be as low as reasonably achievable (ALARA) to minimize the detriment due to the use of ionizing radiation. However, the dosage should be sufficient to provide acceptable image quality for diagnosis and semi-quantitative analysis within a reasonable amount of acquisition time [13].

The 2017 European Association of Nuclear Medicine (EANM) procedural guideline on PET/CT tumor imaging with ^68^Ga-DOTA-SSA recommends an administered activity range of 100 to 200 megabecquerel (MBq), depending on the characteristics of the PET scanner and patient body weight [14]. However, the impact of body mass or other patient-dependent parameters, such as body mass index (BMI) and lean body mass (LBM) on PET image quality has not previously been investigated for ^68^Ga-DOTA-SSA PET/CT.

Several studies have investigated the impact of patient habitus on ^18^Fluorine-fluoro-2-deoxyglucose (^18^F-FDG) PET/CT image quality. Geismar et al. [15] suggested adjusting the linear dose regimen with higher ^18^F-FDG activity per kilogram (kg) of body mass or increase PET acquisition time per bed position for obese patients to achieve more constant image quality [16–19]. A second possibility is a linear dose regimen for patients below 75 kg and a quadratic dose regimen for patients above 75 kg. A quadratic dose regimen was proposed by de Groot et al. [20] in a study to optimize the administered ^18^F-FDG activity as a function of a patient-dependent parameter, for example, body mass, BMI, LBM, fat mass (defined by body mass minus the lean body mass), and body mass per body length. This study showed that a quadratic relationship between administered activity, body mass, and acquisition time delivered a more constant PET image quality than a linear dose regimen for ^18^F-FDG. Moreover, in a later study by Menezes et al. [21], body mass was found as best predictor in a univariate analysis. Furthermore, a multivariate analysis in a study by Wickham et al. [12] showed a combination of body mass, sex, and length as best predictors. Based on the results of de Groot et al. [20], the current EANM guideline for tumor imaging with ^18^F-FDG [22] recommends adjusting the activity quadratically to the body mass, but for pragmatic reasons, the guideline also includes a linear activity-body mass relationship.

Previous phantom studies suggest that ^18^F-FDG findings may not necessarily be applicable to ^68^Ga-DOTA-SSA PET because the impact of the larger positron range of 3.5 mm (mean range in water) of Gallium-68 on image quality [23, 24], and the shorter half-life of 68 min that might necessitate a higher injected activity. Furthermore, ^68^Ga-DOTA-SSA tracer biodistribution differs from ^18^F-FDG biodistribution. Therefore, the relationship between patient-dependent parameters and ^68^Ga-DOTA-SSA PET image quality should be investigated. The aim of this study is to investigate the influence of patient size on ^68^Ga-DOTA-SSA PET image quality and to propose a dose regimen that maintains constant image quality and yields sufficient image quality for clinical use.

## Methods

### Patients

For this study, 21 patients (12 men, 9 women; mean age 60.1 ± 10.1 years) that were scheduled for ^68^Ga-DOTA-TATE PET/CT at the Erasmus Medical Center for staging or restaging of NETs, were prospectively included. Patients were equally distributed according to their weight into seven different groups (50-60 kg, 61-70 kg, 71-80 kg, 81-90 kg, 91-100 kg, 101-110 kg, 111-120 kg) of each three patients. Inclusion criteria were diagnosed with or suspicion of a NET. Exclusion criteria were claustrophobia, unable to maintain scan position for 36 min and already known extensive liver involvement. The study was approved by the Medical Ethical Committee of the Erasmus Medical Center, written informed consent was obtained from all the patients, and procedures were in accordance with the Declaration of Helsinki of 1964, as revised in 2013.

### Patient-dependent parameters

Body mass (kg) and body length (m) were measured before tracer injection. Three other parameters were calculated: BMI by dividing body mass (kg) by the square of length (m^2^), body mass per body length by dividing the body mass (kg) by the body length (m), and lean body mass (LBM) by the approach of Janmahasatian et al. [25].

### ^68^Ga-DOTA-TATE PET/CT

Patients were prepared according to the standard protocol, they were asked to drink 1L of water during the 2 h prior to injection. When patients used long-acting somatostatin analogues (e.g., Sandostatine LAR, Novartis Pharma BV), PET/CT was planned just prior to the next scheduled monthly dose. ^68^Ga-DOTA-TATE was intravenously injected with an activity of 1.5 MBq/kg (128 ± 32.2 MBq). PET images were acquired 61 ± 3 min after tracer injection in supine position with the arms up on a Siemens Biograph mCT PET/CT scanner (Siemens Healthineers, Erlangen, Germany). According to our local scan protocol, first a whole-body low dose CT was acquired for attenuation correction and localization purposes (120 kV, quality reference mAs 40, rotation time 0.5 s, pitch of 0.8 mm, slice thickness of 3 mm; reconstructed slice thickness 3 mm and a Siemens B19f low dose for emission computed tomography kernel). Directly after the low-dose CT, PET acquisition started in list mode, with an acquisition time of 6 mbp, using 6 to 7 bed positions per patient (from skull base to inguinal region).

Prior to image reconstruction, the acquired list-mode PET data were randomly sampled using an in house developed Python script to simulate PET scans of 1, 2, 3, 4, 5, and 6 mbp, similar to the method used by Halpern et al. [16, 17]. All scans were corrected for scatter and attenuation using the low dose CT and reconstructed using ordered subset expectation maximization (OSEM) with point spread function (PSF) recovery, time of flight (TOF), 3 iterations, 21 subsets, a 3 mm Gaussian post reconstruction filter on a matrix of 200 × 200 with a pixel size of 4.1 × 4.1 mm.

### Image analysis

A volume of interest (VOI) was placed in a lesion-free homogeneous part of the right liver lobe (diameter 3 cm) using the Hermes Hybrid viewer 2.6D software (Hermes Medical Solutions, Stockholm, Sweden). The VOIs were placed at least 1 cm from the edge of the liver to avoid partial volume effects. For each patient, VOIs were placed at the exact same location throughout all 6 PET reconstructions. As a measure of image quality, the signal-to-noise ratio (SNR) within the VOI was calculated by dividing the liver SUVmean (liver biodistribution) by the standard deviation (SD). The 6 mbp PET reconstruction served as the reference for comparing the SNR between reconstructions.

PET image quality depends on the time per bed position and the amount of administered activity. The dose-time product (DTP (MBq·min)) is the product of these parameters. By assuming Poisson statistics, noise increases with the square root of the signal, the SNR can be normalized (SNRnorm (MBq·min)^−1/2^)) for the administered activity and scan time per bed position [20]. The SNRnorm is assumed to be independent of scan time and administered activity.

1 $$ \mathrm{SNRnorm}=\frac{\kern0.75em \mathrm{SNR}}{\sqrt{\mathrm{DTP}}} $$

To investigate the relation between patient-dependent parameters and PET image quality, the following patient-dependent parameters were correlated with the mean SNRnorm: body mass, length, BMI, body mass per body length, and LBM. The patient-dependent parameter demonstrating the highest correlation was selected to derive an optimized dose regimen.

In order to compare semi-quantitative measurements between all reconstructions, standardized uptake value (SUV) measurements were obtained of one lesion in each patient with a suspicious lesion located centrally in the abdomen. VOI-based SUV measurements were performed. SUVmax was measured by the voxel with the highest pixel value in the VOI, SUVpeak by the 1 cm^3^ with the highest pixel values inside the VOI [26], and SUVmean was determined using a region growing algorithm with a 50% threshold of SUVmax [22]. These VOIs were placed at the exact same position throughout all PET reconstructions.

All anonymized PET data (21 patients, 5 reconstructions per patient) were presented to three experienced nuclear medicine physicians, for visual assessment of image quality. They were blinded for clinical data.

Reconstructions were presented in the following order to the physicians: 2, 3, 4, 5, and 6 mbp, to avoid lesion recognition bias that might be introduced by viewing images with higher count statistics prior to images with lower count statistics. Each physician scored all reconstructions subjectively for visual diagnostic image quality using the four-point scoring scale from Halpern et al. [17]: non-diagnostic (0), poor (1), moderate (2), or good (3). The median of the three scores was taken as a measure of subjective image quality for each reconstruction.

For lesion detectability, the readers recorded the number of SSTR positive lesions in neck/thorax, liver, abdomen (without liver), and skeleton for each reconstruction in the following categories: 0 lesions (0), 1 lesion (1), 2 lesions (2), 3-5 lesions (3), 6-10 lesions (4), and > 10 lesions (5), yielding 252 scores per mbp. In concordance with the SNR approach, lesion detectability was compared between all reconstructions with the 6 mbp as a reference.

### Statistical analysis

All statistical analyses were performed using IBM SPSS statistics version 24. To test for significant differences in liver biodistribution (liver SUVmean), image quality (SNR and SNRnorm), and lesion quantification (SUVmax, SUVmean, and SUVpeak) in the 1 to 6 mbp reconstructions, a repeated measures analysis of variance (ANOVA) (*α* = 0.05) (including Mauchly’s test of sphericity with a Greenhouse-Geisser correction for non-sphericity) or a non-parametric Friedman test (*α* = 0.05) after testing the data for normality by a Kolmogorov Smirnov test was performed. The Bonferroni multiple comparison post hoc test or the non-parametric post hoc Dunn-Bonferroni test was used to identify significant differences between 1-5 mbp reconstructions and the 6 mbp reconstruction.

The coefficient of determination (*R*^2^) was used to select the best patient-dependent parameter after correlation of each parameter with SNRnorm. Similar to the Groot et al., curve fitting was applied to the parameters by using a power function (Eq. 2):

2 $$ {\mathrm{SNR}}_{\operatorname{norm},\mathrm{fit}}=a\ {p}^{-d},\kern2.5em $$

where *a* and *d* are fit parameters and *p* is the patient-dependent parameter. The relative error between SNRnorm and SNRfit was calculated for each data point using (SNRfit−SNRnorm)/SNRfit × 100%, to perform One-Way ANOVA test (*α* = 0.05) to determine significant differences between the standard deviation of the relative error distribution of the parameter fit with the highest *R*^2^ and the other parameter fits [20].

To compare SNR between the median visual scores, a Kruskal-Wallis test (*α* = 0.05) post hoc Dunn-Bonferroni test was performed. The lesion detectability of all reconstructions was compared with the 6 mbp by using a non-parametric Friedman test (*α* = 0.05) and a post hoc Dunn-Bonferroni test. Interobserver agreement for visual score and lesion detectability was quantified by Fleiss’ kappa (*휅*) for multiple raters.

Combining Eq. 1 and 2, and substituting body mass (m) for the patient-dependent parameter results in the following expression for the DTP (MBq·min):

3 $$ \mathrm{DTP}=\frac{{\mathrm{SNR}}^2}{a^2}\times {m}^{2d}, $$

where SNR is the acceptable noise level that was determined in the image analysis. Above this SNR value, all scans scored as either moderate or good.

## Results

### Patient-dependent parameters

The measurements and calculations of the patient-dependent parameters are displayed in Table 1.

**Table 1:** Patient-dependent parameters

Parameters	Mean ± SD	Range
Body mass (kg)	86.5 ± 19.7	55–124
Body length (m)	1.74 ± 0.1	1.64–1.88
BMI (kg/m^2^)	28.3 ± 5.9	20.2–37.7
Body mass/body length (kg/m)	49.5 ± 10.6	33.3–69.3
Lean body mass (kg)	57.4 ± 11.7	37.2–76.4

### Image quality

**Fig. 1:**
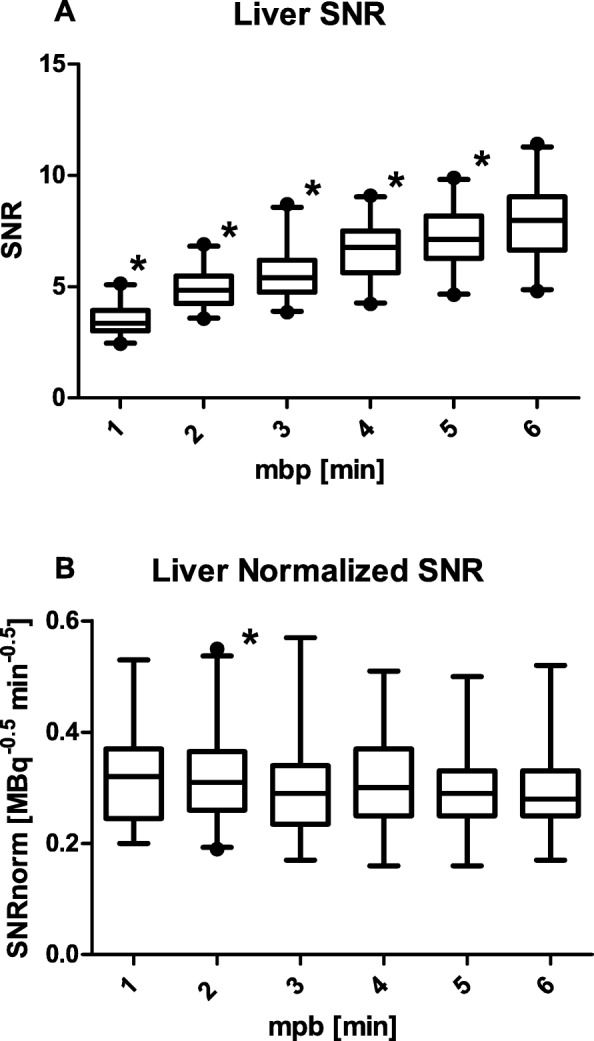
Boxplots of the SNR (**a**) and the SNRnorm (**b**) against the mbp. Whiskers specify median, minimum, and maximum. Significant differences compared with 6 mbp (**a**, *p* < 0.001; **b**, *p* < 0.05) are indicated with an asterisk

A repeated measures ANOVA (*F* (2.95, 1.79) = 0.27 *p* = 0.85) and additional post hoc test with Bonferroni correction (*p* > 0.05) revealed no significant differences in liver SUVmean for 1-5 mbp reconstructions compared with the 6 mbp reconstruction. Figure 1 demonstrates the SNR and SNRnorm for the 1-6 mbp reconstructions. Repeated measures ANOVA determined the mean SNR (*F (*1.67, 29.76) = 180.54, *p* < 0.001) and mean SNRnorm (*F* (1.95, 38.96) = 5.54, *p* = 0.008) and showed significant differences in SNR and SNRnorm. The additional post hoc tests using the Bonferroni correction revealed a significantly lower (*p* < 0.001) mean SNR for all reconstructions compared with the 6 mbp as demonstrated in Fig. 1a. After normalization for administered activity and time per bed position, the post hoc test with Bonferroni correction showed no significant difference in mean SNRnorm (*p* > 0.05) with the 6 mbp, except for 2 mbp (*p* = 0.016) as shown in Fig. 1b.

**Fig. 2:**
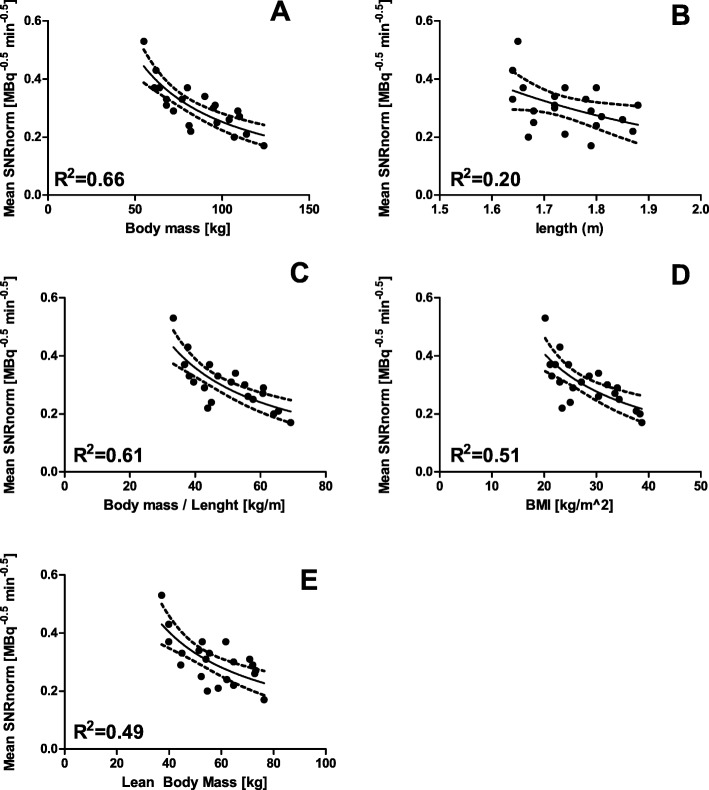
Non-linear fits of the mean SNRnorm ((MBq·min)^−1/2^) versus body mass (**a**), length (**b**), body mass/length (**c**), BMI (**d**), and LBM (**e**). The dashed lines represent the 95% confidence intervals of the fits

After normalization of SNR, the mean of SNRnorm over all the reconstructions was taken and correlated with the described patient-dependent parameters. As can be seen in Table 2, the linear fit of body mass with mean SNRnorm shows the highest coefficient of determination (*R*^2^ = 0.60). The non-linear fits of the mean SNRnorm with the patient-dependent parameters are illustrated in Fig. 2. The fit parameters for body mass were determined at *a* = 19.6 and *d* at 0.95 and *R*^2^ = 0.66. The 95% confidence interval for parameter *d* was computed at 0.62-1.27. A One-Way ANOVA test revealed only a significant difference between the fit of body mass and the fit of length (*F* (1, 40) = 235.8 *p* < 0.001).

**Table 2:** Coefficients of determination obtained from the linear correlations between the mean SNRnorm and the patient-dependent parameters

Patient-dependent parameter	*R*^2^
Body mass (kg)	0.60
Body mass/length (kg/m)	0.57
LBM (kg)	0.42
BMI (kg/m^2^)	0.50
Length (m)	0.18

### Lesion quantification

A SSTR-positive lesion located centrally in the abdomen was found in 16 patients. SUV measurements of these lesions are presented in Table 3. A Friedman test was performed for SUVmean and showed no significant differences between the reconstructions (*χ*2 (5) = 3.49, *p* = 0.625). The same test was applied for SUVmax (*χ*2 (5) = 17.86, *p* = 0.03). The additional Dunn-Bonferroni post hoc test revealed no significant difference between 1-5 mbp and 6 mbp reconstructions (*p* > 0.05). Also, a repeated measures ANOVA reported no significant difference for SUVpeak comparing the 1-5 mbp with the 6 mbp reference reconstruction (*F* (1.16, 17.45) = 0.84, *p* = 0.390).

**Table 3:** Lesion semi-quantification

Reconstruction	SUVmean (mean ± SD)	SUVmax (mean ± SD)	SUVpeak (mean ± SD)
1 mbp	31.8 ± 34.4	47.4 ± 49.7	33.5 ± 35.3
2 mbp	29.8 ± 28.0	44.0 ± 37.9	32.2 ± 29.1
3 mbp	29.8 ± 29.5	43.0 ± 40.3	32.0 ± 30.4
4 mbp	30.0 ± 29.6	42.5 ± 40.0	32.0 ± 30.5
5 mbp	29.7 ± 29.6	41.6 ± 39.5	31.6 ± 30.5
6 mbp	30.0 ± 29.3	42.1 ± 39.5	32.0 ± 30.3

### Visual image quality

Table 4 gives an overview of the visual scores for each reconstruction per reader. Overall, there was a fair agreement between the three readers according to the calculated Fleiss *휅* (0.34 (*p* < 0.001)), 95% CI (0.26-0.42). As can be seen, only four scans were scored as non-diagnostic by only one reader. Therefore, the median score of these individual visual scores revealed no non-diagnostic score.

**Table 4:** Visual image quality and interobserver agreement

PET image quality	Non-diagnostic			Poor			Moderate			Good		
Reconstruction	Reader			Reader			Reader			Reader		
	1	2	3	1	2	3	1	2	3	1	2	3
2mbp	**-**	**-**	4	5	15	14	14	6	2	2	**-**	1
3mbp	**-**	**-**	**-**	**-**	4	10	7	11	9	14	6	2
4mbp	**-**	**-**	-	-	-	3	-	8	8	21	13	10
5mbp	**-**	**-**	-	-	-	-	-	1	2	21	20	19
6mbp	**-**	**-**	-	-	-	-	-	-	-	21	21	21

Overall interobserver agreement Fleiss *휅* of 0.34 (*p* < 0.001), 95% CI (0.26-0.42)

**Fig. 3:**
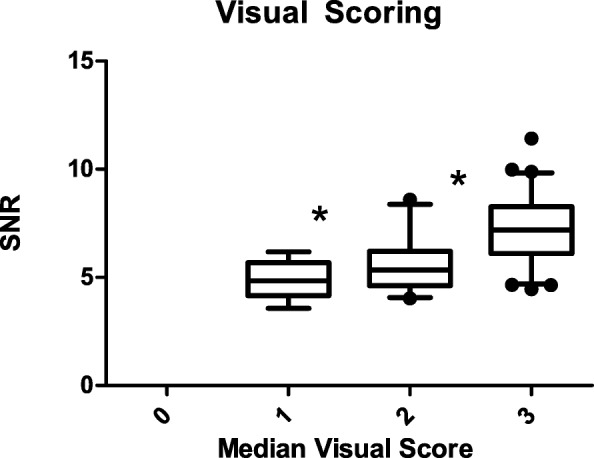
Boxplots of SNR against median visual score (non-diagnostic (0), poor (1), moderate (2), or good (3)) with median and ranges. Significant differences between poor-good and moderate-good (*p* < 0.001) are marked with an asterisk

A Kruskal-Wallis test for comparing the other median scores to the SNR showed highly significant differences (*χ*2 (2) = 40.77, *p* < 0.001) in mean ranks of SNR between visual score categories. A post hoc Dunn-Bonferroni test revealed a strong significant difference between poor and good (*p* < 0.001) and moderate and good (*p* < 0.001) image quality. Between poor and moderate, no significant difference (*p* = 0.405) was found (Fig. 3). All scans with a SNR higher than 6.2 were scored as either moderate or good.

### Lesion detectability

**Fig. 4:**
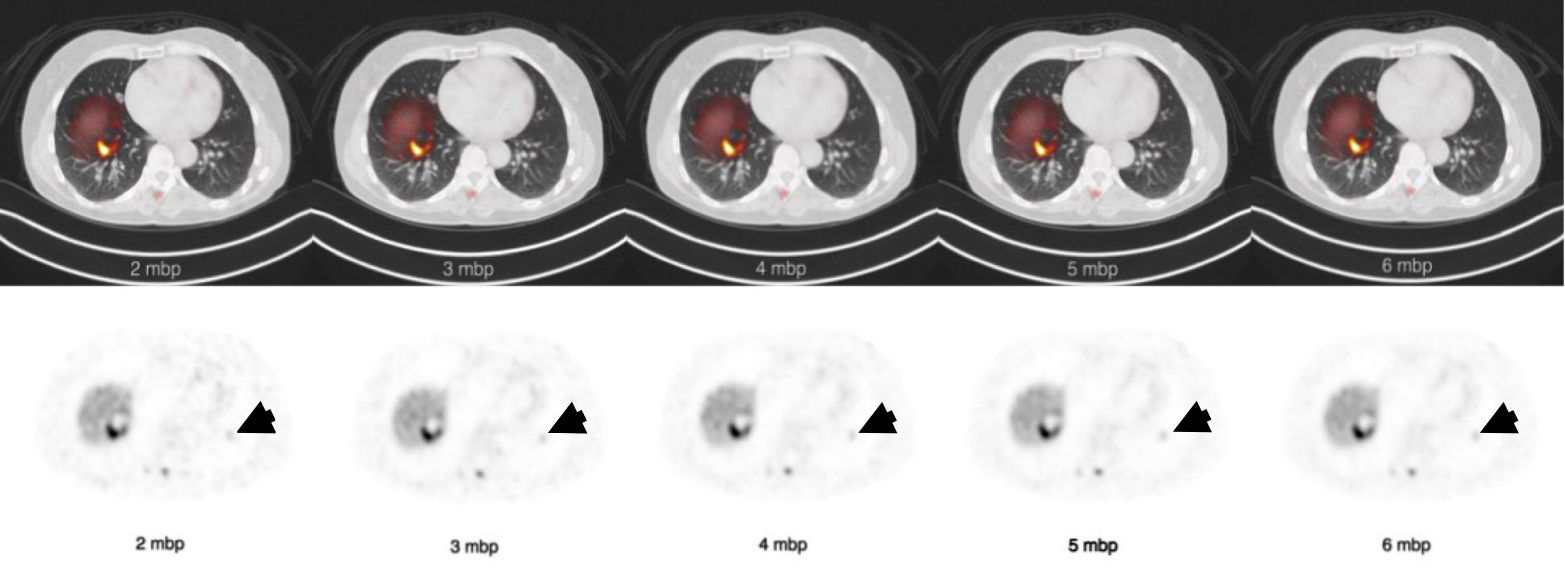
Example of transversal PET/CT fusion and PET only images. The images show multiple lesions including one lesion in the liver and one lesion with low uptake in the left basal lung (arrow). The lung lesion was detected on 3-6 mbp and was missed on 2 mbp. All other lesions were detected on all mbps

The results of the lesion detectability assessment are summarized in Table 5. It can be appreciated that the 2 mbp has a significant lower lesion detectability rate than the other reconstructions compared with the 6 mbp. In Fig. 4, a lesion located in the lower part of the left lung is shown for a single patient. A Friedman test (*χ*2 (4) = 122.54, *p* < 0.001) with an additional Dunn-Bonferroni post hoc test confirmed that only the 2 mbp had a significant difference (*p* = 0.007) in lesion categories with the 6 mbp reference reconstructions. Furthermore, the table shows that the interobserver agreement was almost perfect for 2 mbp (Fleiss *휅* = 0.81) and substantial (Fleiss *휅* = 0.79-0.74) for the other reconstructions.

**Table 5:** Number of body regions that were scored for lesion detectability by three readers for each reconstruction and interobserver agreement

Number of lesions	Reconstruction				
	2 mbp*	3 mbp	4 mbp	5 mbp	6 mbp
0	139	133	128	127	126
1	29	25	30	31	29
2	17	19	11	9	11
3-5	23	28	35	34	35
6-10	19	21	21	21	20
>10	25	26	27	30	31
Interobserver agreement	0.81	0.79	0.76	0.74	0.74
Fleiss *휅*					

Significant differences compared with 6 mbp (*p* < 0.001) are marked with an asterisk

### Constant image quality

Using an SNR of 6.2 as an acceptable noise level in the PET images and the fitted values for *a* (19.6) and *d* (0.95), the DTP needed for a more constant image quality as a function of body mass is given by Eq. 4:

4 $$ \mathrm{DTP}=0.10\times {m}^{1.9} $$

## Discussion

The aim of this study was to investigate the influence of patient-dependent parameters on ^68^Ga-DOTA-TATE PET image quality and to propose a dose regimen that maintains constant image quality while providing sufficient image quality for clinical use. To the best of our knowledge, this study is the first that investigates the relationship between patient-dependent parameters and ^68^Ga-DOTA-TATE PET image quality.

The image quality analysis is based on liver SUVmean, this value remained stable throughout all reconstructions. As expected, the image quality as measured by the SNR in the liver increased with prolonged acquisition time (Fig. 1a). After normalizing the SNR by the method of de Groot et al. [20], the normalized image quality of 1 and 3-5 mbp shows no significant difference compared with the 6 mbp reconstruction (Fig. 1b). Therefore, we can conclude normalizing the SNR is a valid method to quantify image quality independent of mbp.

In this study we found no significant changes in SUVs of lesions with a SUVmean > 5.2 relative to the SUVmean (30.0 ± 29.3), SUVmax (42.1 ± 39.5), and SUVpeak (32.0 ± 30.3) of 6 mbp reconstruction after reducing PET acquisition time (Table 3). These results coincide with the conclusions of ^18^F-FDG studies by Goethals et al. [27] and Garcia-Velloso et al. [28], which also reported no significant changes in SUV measurements between 1 mbp and longer acquisition times. Although quantification of lesions with a lower uptake or low target to background ratio could be affected by reduced count statistics, but this was not investigated in our study. Furthermore, we have used a 3-mm Gaussian post reconstruction filter together with a PSF recovery reconstruction that is suitable for detecting smaller lesions. It is known that these reconstructions are difficult to use for harmonized quantification in a multi-center setting [29, 30], since they show a higher recovery for smaller lesions and suffer from possible overestimation of the SUVmax due to the Gibb’s artifact [29]. For ^18^F-FDG, a substantial larger filter of 7 mm was proposed to allow PSF to fall within the limits currently set by the EANM Research Ltd. (EARL) ^18^F-FDG PET/CT accreditation program [30], possibly at the expense of decreased lesion detectability. More research is needed for harmonized quantification in ^68^Ga-DOTA-SSA imaging.

Correlation of patient-dependent parameters revealed that body mass versus mean SNRnorm had the highest coefficient of determination (*R*^2^ = 0.60) for the linear fit (Table 2) and showed the highest *R*^2^ (0.66) for the non-linear fit (Fig. 2). However, the other fits for the patient dependent parameters were not significantly different to the body mass fit, except for patient length. Since body mass is easy to use in practice, we used body mass to determine the optimal dose regimen. The non-linear fit with body mass clarified 66% of variability in mean SNRnorm between patients. The remaining 34% variability in SNRnorm was not clarified in this study. This variability might be caused by the difference in patient habitus, which is not captured in the single body mass parameter. Although, no difference in liver biodistribution (mean liver SUVmean of 6 mbp 5.8 ± 1.3) between the reconstructions was found. Variation could also be caused by possible (unknown) liver inhomogeneities within the VOI. Nevertheless, the clarified variability is slightly lower but comparable with findings by de Groot et al. [20] and Menezes et al. [21]. In both ^18^F-FDG studies, body mass also has the highest *R*^2^ with respectively SNRnorm (*R*^2^ = 0.77, Biograph mCT with OSEM3D + PSF + TOF reconstruction) and the normalized coefficient of variation (*R*^2^ = 0.86, Biograph trueV with OSEM3D + PSF reconstruction). However, they found that body mass was significantly superior to some (de Groot et al.) and all (Menezes et al.) other parameters, and these studies included more (respectively 62 and 58) patients at random, but relatively few patients at the extremes (< 60 kg and > 100 kg). The small number of included patients is a major limitation of this study. However, we have sampled different weights as equally as possible through carefully selecting patients into the described weight categories to optimize the weight distribution. The small number of patients included in the current study results in a broad 95% confidence interval for parameter *d* (0.62–1.27). However, it can be concluded that a non-linear dose regimen is needed to achieve a more constant image quality, since a linear dose regimen would require a value of 0.5 for parameter *d* (Eq. 3). A number of studies [12, 19, 21, 31, 32] used noise equivalent count rate (NECR) data as a measure of PET image quality. The NECR method is proposed to be more objective [12, 31, 32], as the liver SNR method might also be affected by variation in liver metabolism and reconstruction parameters. Menezes et al. [21] used also NECR data, and also found body mass as the parameter with the strongest correlation (*R*^2^ = 0.72). Furthermore, using the NECR method, Wickham et al. [12] developed a multivariate fit based on patient sex, height, and weight for the Biograph mCT.

The visual assessment of image quality revealed a fair agreement between the three experienced readers. Comparing their median visual score to SNR resulted in an SNR of 6.2 for sufficient image quality for clinical use. A possible bias in visual scoring may have been introduced by sequential scoring of the images from high to low noise levels. In two previous ^18^F-FDG studies [20, 32], SNRs of 9.6 and 10 respectively were determined as acceptable image quality. A reason for the lower SNR value required for ^68^Ga-DOTA-TATE could be the higher tumor-to-background ratio [33], due to the different biodistribution of the tracer compared with ^18^F-FDG [34]. Hence, findings for optimized image quality in ^18^F-FDG imaging should not be translated directly to ^68^Ga-DOTA-TATE PET imaging.

The required DTP for a more constant image quality as a function of body mass is given in Eq. 4. It is assumed that for clinical used dose regimens, the administered activity and mbp can be chosen independently. However, increasing the activity rather than the mbp will have a negative impact on image quality, especially for higher count rates, due to scanner and object dependent non-linear NECR curves. Therefore, Machado et al. proposed to increase the mbp for patients > 90 kg rather than increasing the injected activity. They also observed a lower value for the parameter *d* for smaller amounts of injected ^18^F-FDG that was attributed to the difference in NECR. Furthermore, enabling PSF and TOF affected the value of parameter *d* [20, 30]. Machado et al. found a linear relationship between body mass and DTP using a PSF reconstruction and a Gaussian post reconstruction filter of 7 mm. The optimal dose regimen in this study scales the body mass to the power of 1.9. This is within the same range as found in other studies (1.5-2.4) [12, 20, 30]. Values are difficult to compare directly due to the use of a non-TOF scanner (Machado et al.), differences in number of iterations (2-3) and a different Gaussian filter (2_5 mm).

Both NECR and liver SNR do not provide a complete measure of image quality and do not yield adequate information about image resolution and contrast recovery. Since both are of key importance for lesion detectability, the number of lesions observed by the physicians was analyzed. The lesion detectability did not change for lower mbp reconstructions, only the 2 mbp reconstruction revealed a significantly (*p* = 0.007) lower lesion detectability compared with the 6 mbp reference reconstructions. Similar to lesion semi-quantification, lesion detectability was found to be still reliable while decreasing DTP. As this study included patients with extensive disease it was not possible to count every lesion separately in all patients on all reconstructions. Therefore, we were limited to categorization of the number of lesions. Consequently, a difference of one lesion between reconstructions might occur within the same lesion category in patients with multiple lesions in a certain body region. This differs from ^18^F-FDG and other ^68^Ga-DOTA-SSA studies where the approach of counting the total amount of lesions was used [8–10, 16, 17]. Another limitation concerning lesion detectability is recognition bias that can occur by the serial viewing of the reconstructions from short to longer acquisition times.

Simulated shorter acquisition times did not affect SUVs of lesions (SUVmean > 5.2) and lesion detectability did not significantly differ for reconstructions longer than 2 mbp. However, from the visual assessment analysis an SNR of 6.2 was determined acceptable. This requires minimal 4 mbp (Fig. 1a) acquisition time for the dose regimen used in this study. Hence, the subjective assessment of image quality is the limiting factor for reducing DTP.

As body mass has the greatest influence on the image quality of ^68^Ga-DOTA-TATE PET scans, a dose regimen based on the patient-dependent parameter body mass (Eq. 4) is needed to maintain constant image quality throughout the patient population. With an acquisition time of 3 mbp, the activity range in the study population would be 68 MBq to 317 MBq for patients weighing 55 to 124 kg. The current EANM guideline [14] recommends an activity of at least 100 MBq and up to 200 MBq, independent of patient habitus. This guideline may result in unnecessarily high activity for patients with low body mass (< 68 kg) or an unacceptable poor image quality for patients with high body mass (> 98 kg) according to our dose regimen in case of an acquisition time of 3 mbp.

The proposed dose regimen is based on DTP (MBq·min) instead of activity (MBq). The dose regimen also allows for an increase in scan time to compensate for a lower activity due to the decreasing elution output of a ^68^Ge/^68^Ga generator (half-life 270.8 days). This enables a more cost-effective use of an expensive ^68^Ge/^68^Ga generator.

## Conclusion

Body mass can be used to predict ^68^Ga-DOTA-TATE PET image quality. The proposed non-linear dose regimen based on body mass standardizes the image quality while maintaining sufficient image quality for diagnosis.

## Data Availability

The data supporting our findings are available upon request.

## Abbreviations

^111^In-Octreoscan: ^111^In-pentetreotide (^111^In-DTPA^0^-octreotide)
^177^Lu-DOTA-TATE: ^177^Lutetium-DOTA-tyr3-Octreotate
^18^F-FDG: ^18^Fluorine-fluorodeoxyglucose
^68^Ga-DOTA-NOC: ^68^Gallium-DOTA-1-Nal3-Octreotide
^68^Ga-DOTA-TATE: ^68^Gallium-DOTA-Tyr3-Octreotate
^68^Ga-DOTA-TOC: ^68^Gallium-DOTA-D-Phe1-Tyr3-Octreotide
ALARA: As low as reasonably achievable
ANOVA: Analysis of variance
BMI: Body mass index
DTP: Dose-time-product
EANM: European Association of Nuclear Medicine
EARL: EANM Research Ltd.
kg: Kilogram
mbp: Minutes per bed position
MBq: Megabequerel
NECR: Noise equivalent count rate
NET: Neuroendocrine tumour
OSEM: Ordered subset expectation maximization
PET: Positron emission tomography
PET/CT: Positron emission tomography/computed tomography
PRRT: Peptide receptor radionuclide therapy
PSF: Point spread function
SD: Standard deviation
SNR: Signal-to-noise ratio
SNRnorm: Normalized signal-to-noise ratio
SPECT: Single photon emission computed tomography
SSA: Somatostatin analogues
SSTR: Somatostatin receptor
SUVmax: Maximum standardized uptake value
SUVmean: Mean standardized uptake value
SUVpeak: Peak standardized uptake value
TOF: Time of flight
VOI: Volume of interest
